# Placental Syncytium Forms a Biophysical Barrier against Pathogen Invasion

**DOI:** 10.1371/journal.ppat.1003821

**Published:** 2013-12-12

**Authors:** Varvara B. Zeldovich, Casper H. Clausen, Emily Bradford, Daniel A. Fletcher, Emin Maltepe, Jennifer R. Robbins, Anna I. Bakardjiev

**Affiliations:** 1 Department of Pediatrics, University of California, San Francisco, San Francisco, California, United States of America; 2 Program in Microbial Pathogenesis and Host Defense, University of California, San Francisco, San Francisco, California, United States of America; 3 Department of Bioengineering and Program in Biophysics, University of California, Berkeley, Berkeley, California, United States of America; 4 Biomedical Sciences Program, University of California, San Francisco, San Francisco, California, United States of America; 5 Department of Biology, Xavier University, Cincinnati, Ohio, United States of America; Boston Children's Hospital, United States of America

## Abstract

Fetal syncytiotrophoblasts form a unique fused multinuclear surface that is bathed in maternal blood, and constitutes the main interface between fetus and mother. Syncytiotrophoblasts are exposed to pathogens circulating in maternal blood, and appear to have unique resistance mechanisms against microbial invasion. These are due in part to the lack of intercellular junctions and their receptors, the Achilles heel of polarized mononuclear epithelia. However, the syncytium is immune to receptor-independent invasion as well, suggesting additional general defense mechanisms against infection. The difficulty of maintaining and manipulating primary human syncytiotrophoblasts in culture makes it challenging to investigate the cellular and molecular basis of host defenses in this unique tissue. Here we present a novel system to study placental pathogenesis using murine trophoblast stem cells (mTSC) that can be differentiated into syncytiotrophoblasts and recapitulate human placental syncytium. Consistent with previous results in primary human organ cultures, murine syncytiotrophoblasts were found to be resistant to infection with *Listeria monocytogenes* via direct invasion and cell-to-cell spread. Atomic force microscopy of murine syncytiotrophoblasts demonstrated that these cells have a greater elastic modulus than mononuclear trophoblasts. Disruption of the unusually dense actin structure – a diffuse meshwork of microfilaments - with Cytochalasin D led to a decrease in its elastic modulus by 25%. This correlated with a small but significant increase in invasion of *L. monocytogenes* into murine and human syncytium. These results suggest that the syncytial actin cytoskeleton may form a general barrier against pathogen entry in humans and mice. Moreover, murine TSCs are a genetically tractable model system for the investigation of specific pathways in syncytial host defenses.

## Author Summary

Infection of the placenta can lead to pregnancy complications as well as fetal and maternal disease and death. We developed a novel system to study placental infections using murine fetal placental progenitor cells and the bacterial pathogen *Listeria monocytogenes*. In the mature placenta fetal progenitor cells fuse to form a large surface (syncytium) that is bathed in maternal blood and mediates nutrient and gas exchange between maternal and fetal circulation. We found that the syncytium resists physical deformation, and that its unusual cytoskeletal organization contributes to its elasticity. Weakening of its elastic properties correlated with increased susceptibility to infection. Our study presents a novel system to study placental infections, and provides new insights into the nature of the placental barrier.

## Introduction

Intrauterine infection is associated with pregnancy complications such as preterm labor [1], which affects 10% of all live births [2]. All of the hematogenous placental microbes have at least partially intracellular life cycles [3]. Among these is *L. monocytogenes*, a facultative intracellular bacterium that causes foodborne disease in humans and other mammals. The relative risk of listeriosis is ∼115-fold higher in pregnant women compared to non-pregnant women of reproductive age [4]. The Centers for Disease Control reported 1,651 cases in the US during 2009–2011, of these 227 (14%) were pregnancy-associated [5]. *L. monocytogenes* triggers preterm labor and spreads to the fetus; the neonatal case-fatality rate is 22–45% [6]–[10]. Thus, pregnancy-associated listeriosis is a severe but rare disease. However, *L. monocytogenes* is ingested frequently by healthy adults [11]. Thus, it seems reasonable to hypothesize that the maternal-fetal interface forms an extremely effective barrier against infection. Perhaps the etiology of preterm labor is multifactorial even in cases of documented intrauterine infection. Indeed, recent evidence suggests that a combination of host genetic factors and bacterial products triggers preterm labor [12].

The placenta is a transient chimeric organ composed of maternal and fetal cells, and serves two major roles in the course of gestation: to nourish and to protect the fetus. The placenta must protect the fetus both from pathogens and from rejection by the maternal immune system [13], [14], which results in a unique immunological environment. The prevailing notion has been that fetal tolerance mechanisms create an immune-privileged site prone to infection [15]; however, recent evidence suggests that the placenta has effective innate defenses against microbial invasion and replication [3], [16], [17].

The placenta establishes its complex structure throughout the course of gestation: Fetal trophoblasts differentiate into several specialized cell types that perform critical placental functions [18]. Invasive trophoblasts penetrate the uterine lining (decidua) at the implantation site and remodel maternal arterioles to facilitate maternal blood flow into the intervillous space in humans or the labyrinth in mice. Inside these compartments, maternal blood bathes syncytiotrophoblasts (or syncytium, SYN), which mediate nutrient and gas exchange between mother and fetus. Syncytiotrophoblasts form a multinucleated fused surface covered by a dense network of branched microvilli that spans an area of 12 m^2^ at the end of human gestation [19]. While the structural organization of the maternal-fetal interface differs between mouse and human (villous versus labyrinthine placenta), the syncytium separates fetal and maternal blood in both species and serves analogous functions.

We have previously shown that the main interface between mother and fetus, the human syncytium, is resistant to invasion by *L. monocytogenes* [20]. Further, we and others have found that the syncytium is resistant to viral and protozoan infection as well [17], [21], [22]. Therefore, we wanted to explore underlying mechanisms of syncytial resistance and identify factors that could make the syncytium more susceptible to infection, and possibly predispose to infection-triggered pregnancy complications.

One underlying mechanism of its resistance to microbes is the paucity of receptors on its blood-bathed surface [23] and the lack of intercellular junctions; pathogens typically exploit receptors that are components of intercellular junctions to breach epithelial barriers [3], [24]. However, we have also shown syncytial resistance to infection by receptor independent mechanisms including cell-to-cell spread of *L. monocytogenes* from infected macrophages, and direct invasion by *T. gondii*, suggesting that additional mechanisms contribute to syncytial defenses [20], [21]. Syncytiotrophoblasts have an unusually dense cytoskeletal network most likely necessary to support such a laterally vast structure [25]. This led us to hypothesize that syncytiotrophoblasts form a physical barrier to invasion. The dense, branched microvilli on its surface may inhibit pathogen adhesion, and the unusually dense actin network could restrict the physical deformations necessary for pathogen entry.

Primary human tissue can be difficult to obtain for many laboratories, is subject to donor-to-donor variability, and cannot be easily manipulated. Therefore, we turned to mouse trophoblast stem cells that can be differentiated in vitro into syncytiotrophoblasts and mimic the features of human syncytiotrophoblasts [26]. Isolation of mouse trophoblast stem cells (mTSC) is an established process that results in self-renewing cells that can be propagated in culture for many generations [27]. Recent advances in culturing mTSCs without underlying feeder cells [26] have made it suitable for study of placental infection with intracellular pathogens. Murine TSCs can be syncytialized in vitro by chemical inhibition of the ERK/MAP kinase pathway [26], enabling experimental investigation of isolated syncytium in culture. Murine TSCs also open up the possibility to explore the contribution of specific molecular pathways to host defenses via isolation of mTSCs from genetically manipulated mice.

We used syncytialized mTSCs from C57BL/6 mice to investigate the biophysical defense mechanisms of the syncytium. Like previous studies of the human syncytium, we found that the mouse syncytium is relatively resistant to infection by *L. monocytogenes* via two routes – direct invasion and cell-to-cell spread. Biophysical measurements of mouse syncytium with atomic force microscopy (AFM) demonstrated that it has a significantly greater resistance to deformation (elasticity) than mononuclear trophoblasts. Disruption of the actin cytoskeleton decreases syncytial elasticity and correlates with increased bacterial invasion. This finding was confirmed in primary human placental organ cultures, suggesting that the biophysical properties of the syncytium contribute to host defenses in both species.

## Results

### *L. monocytogenes* infects and grows in mTSCs

**Figure 1:**
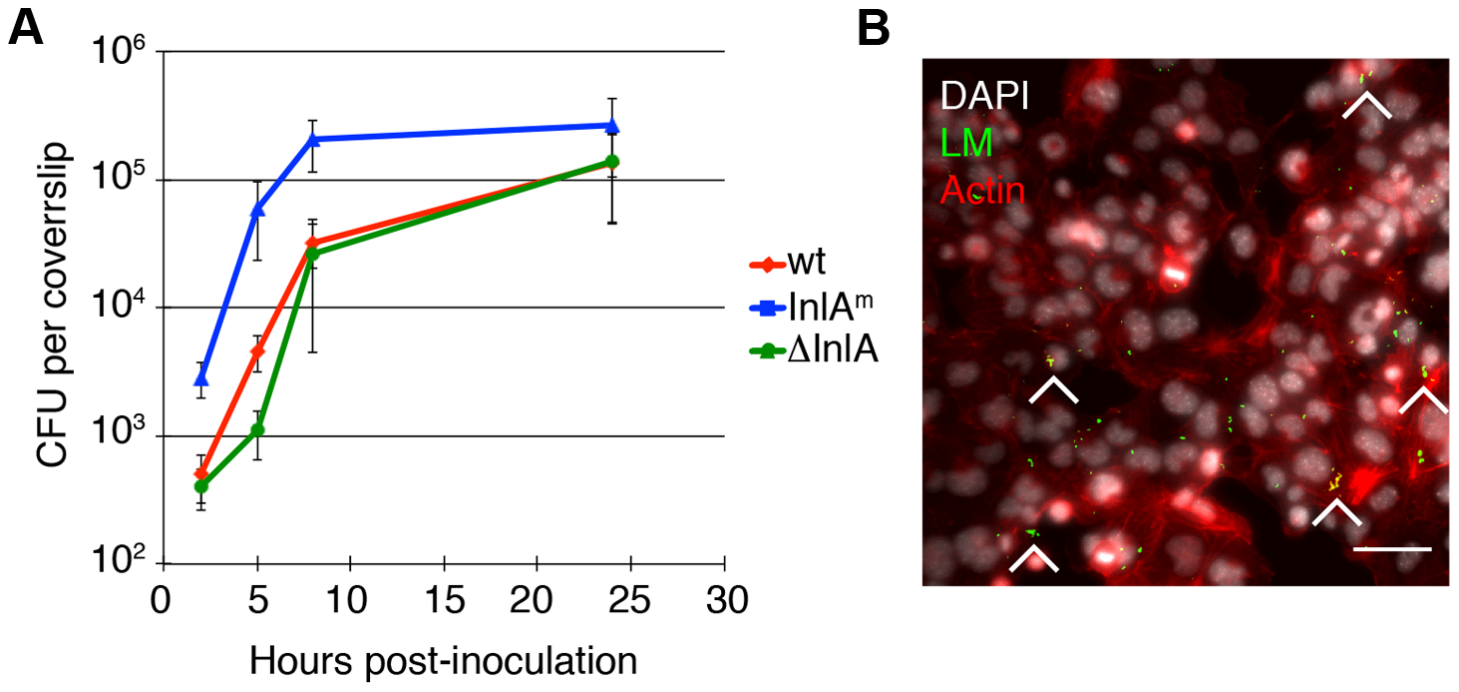
*L. monocytogenes* (LM) invades and replicates in murine trophoblast stem cells. A. Colony forming units (CFU) per coverslip at 2, 5, 8, and 24 hours post-inoculation (p.i.) for the following *L. monocytogenes* (LM) strains were determined: wild type (wt), InlA-deficient (del-InlA), and murinized InlA (InlA^m^). Average CFU per coverslip at 2 hours p.i. for each strain is based on nine independent experiments. Average CFU per coverslip at time points 5, 8, and 24 hours p.i. for each strain are based on three independent experiments. Bars represent standard error. Average CFU at 2 hours for InlA^m^ is 30-fold higher than for wild type and the p-value denotes statistical significance (p = 0.008 by Student's T-test). There is no difference in invasion for wild type versus del-InlA (p = 0.3 by Student's T-test). **B.** Immunofluorescence image of mTSC at 2 hours p.i.: LM is shown in green, nuclei in white, actin in red. Arrowheads point to foci of infection, Bar = 50 um.

Murine TSCs were infected with wild type *L. monocytogenes*, 10403S [28] at a multiplicity of infection (MOI) of 12. Gentamicin was added to the culture medium at 1 hour post-inoculation (p.i.) to eliminate extracellular bacteria. On average 32% of mTSCs were infected with one bacterium at 2 hours p.i., and we observed robust intracellular bacterial replication of *L. monocytogenes* in mTSCs (Fig. 1). The bacterial numbers increased by 64-fold, and the doubling time was 80 min between 2 and 8 hours p.i.

Previous studies have shown that entry into primary human trophoblasts is dependent on the bacterial virulence factor Internalin A (InlA) [29], [30], and that the interaction of InlA with its host cell receptor E-cadherin is species specific [31]. Therefore, we also infected mTSCs with *L. monocytogenes* expressing murinized InlA (InlA^m^), which was engineered for optimal interaction with mouse E-cadherin [32], and with a mutant strain deficient in InlA (del-InlA) [30] (Fig. 1A). Average InlA^m^-expressing bacteria per coverslip at 2 hours p.i. were 30-fold higher than for wild type (p = 0.008 by Student's T-test). There was no difference in the degree of invasion for wild type versus del-InlA. Intracellular growth of each strain was similar in mTSCs with doubling times of 78–80 min between 2 and 8 hours p.i. These results are consistent with a role of InlA in direct invasion of mTSC, E-cadherin expression on mTSCs [26], and species-specificity of InlA/E-cadherin interaction [31].

### Murine syncytiotrophoblasts are resistant to bacterial invasion

**Figure 2:**
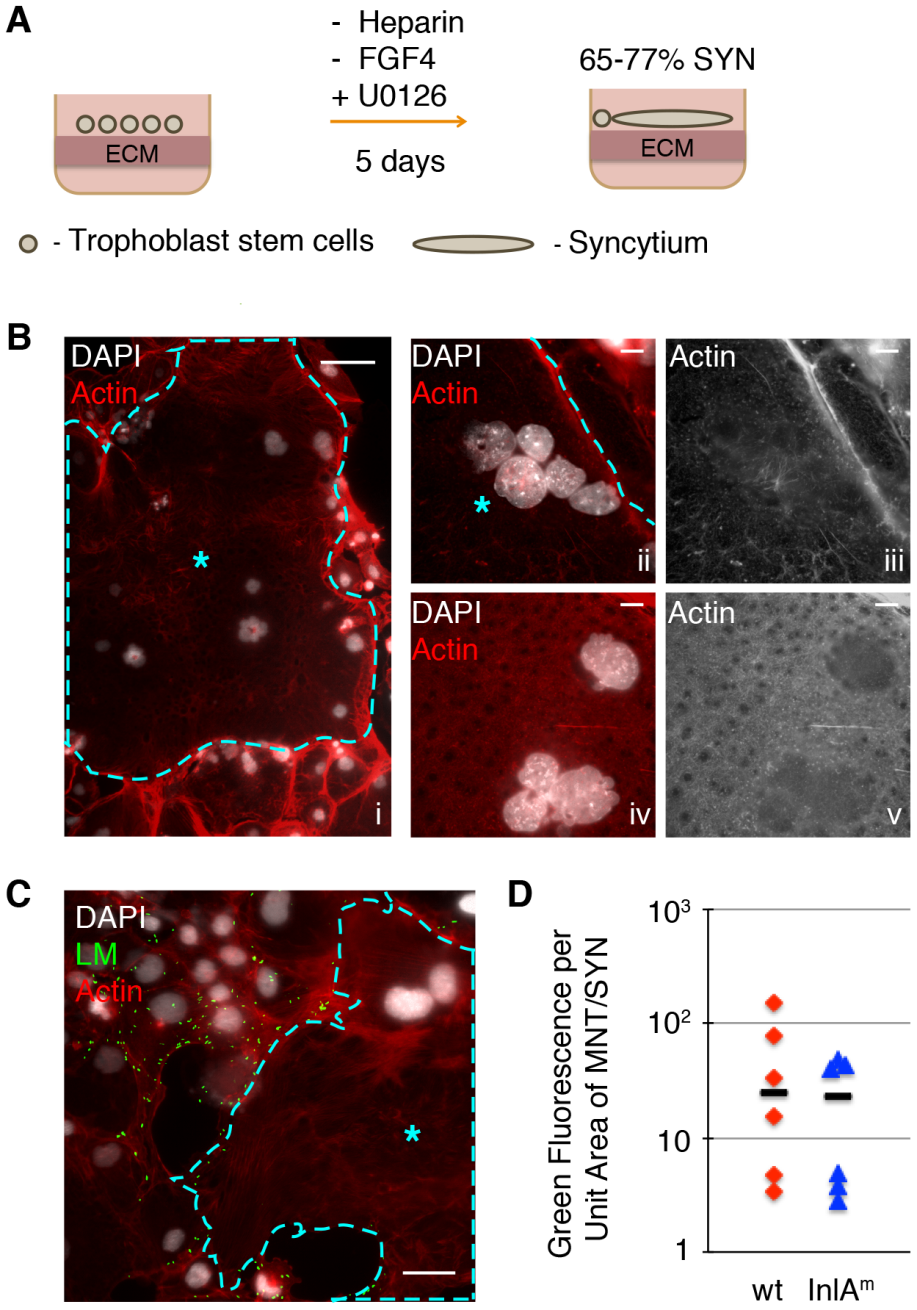
Syncytiotrophoblasts (SYN) derived from murine trophoblast stem cells (TSC) resist direct invasion by *L. monocytogenes* (LM). A. Schematic of differentiation process from TSC to SYN, which results in 65–77% of fused syncytium; the remaining cells in the dish are undifferentiated mononuclear trophoblasts (MNT). **B.** Panel i: Immunofluorescence images of 5-day differentiated multinuclear SYN (outlined and marked by blue star) demonstrates typical clustering of nuclei. Compare to surrounding MNT with stress-fibers, and clear cell boundaries. Bar = 50 um. Panels ii to v show close-up representative examples of actin structure of SYN: a diffuse meshwork of small actin filaments (iii and v show the actin channel of ii and iv respectively). Nuclei are shown in white, Bar = 10 um. **C.** SYN 2 hours p.i. with LM (green) showing resistant syncytial area (outlined and marked by blue star) neighboring infected mononuclear trophoblasts. Nuclei are shown in white; Bar = 50 um. **D.** Quantification of invasion of mononuclear trophoblasts (MNT) versus syncytium with two strains of LM – InlA^m^-expressing and wt. Bacterial invasion at 2 hours p.i. of MNT versus SYN is represented by the ratio of green fluorescence per unit area for each cell type. Each symbol represents the average of green fluorescence per unit area in six random microscopic fields (20×), bar represents median.

Next we characterized infection of murine syncytiotrophoblasts with *L. monocytogenes*. Differentiation of mTSCs into syncytiotrophoblasts was induced by the removal of FGF4 and heparin from the culture medium, and enhanced by addition of MEK inhibitor (Fig. 2A) [26]. Syncytiotrophoblasts were clearly recognized by their unique morphology, specifically the lack of long actin stress fibers and intercellular junctions that encompassed multiple nuclei (Fig. 2B) [25], [26]. As previously described, short actin filaments formed a thick meshwork across syncytial patches. After 5 days under differentiating conditions, syncytiotrophoblasts covered 65% to 77% of the cell culture dish. The remaining area was occupied by mononuclear trophoblast cells that were not terminally differentiated; these contained stress fibers and prominent boundaries (Fig. 2B).

Differentiated TSCs were infected with InlA^m^-expressing *L. monocytogenes*. In addition, wild type *L. monocytogenes* was used to determine effects on invasion that are independent of E-cadherin expression on host cells. Because syncytialization of mTSCs in each well is incomplete, colony-forming units (CFU) would reflect the number of intracellular bacteria in both syncytium and neighboring mononuclear trophoblasts. Therefore, we determined the degree of infection at 2 hours p.i. by immunofluorescence microscopy. We outlined the area of syncytiotrophoblast (Fig. 2C). The number of bacteria was represented by the green fluorescence intensity overlying the area of the syncytiotrophoblast in six random microscopic fields. Infection of mononuclear trophoblasts in the same well was determined by the same method, and the ratio of green fluorescence intensity overlying mononuclear trophoblast versus syncytiotrophoblasts was determined (Fig. 2D). Invasion of syncytiotrophoblasts was lower than invasion of mononuclear trophoblasts by ∼25-fold (Fig. 2D). These findings indicate that murine syncytiotrophoblasts are more resistant to bacterial invasion than mononuclear trophoblasts, and are consistent with previous observations in primary human placental organ cultures. Difference in invasion of mononuclear versus syncytiotrophoblasts was similar for wild type and InlA^m^-expressing *L. monocytogenes*, suggesting that resistance of the syncytium to pathogen entry is not just due to differential E-cadherin expression.

### Syncytiotrophoblasts have greater elasticity than mTSCs

We analyzed the surface resistance to deformation (elasticity) of syncytiotrophoblasts by microrheology with an atomic force microscope (AFM) [33]. For comparison, we measured the elastic modulus of mTSC. We chose undifferentiated mTSC instead of mononuclear trophoblasts, because the partially differentiated mononuclear trophoblasts are a heterogeneous population of cells at varying stages of differentiation towards syncytium. Given the large variability between samples in this system we decided to measure the extremes of the spectrum: undifferentiated mTSC versus terminally differentiated syncytium.

**Figure 3:**
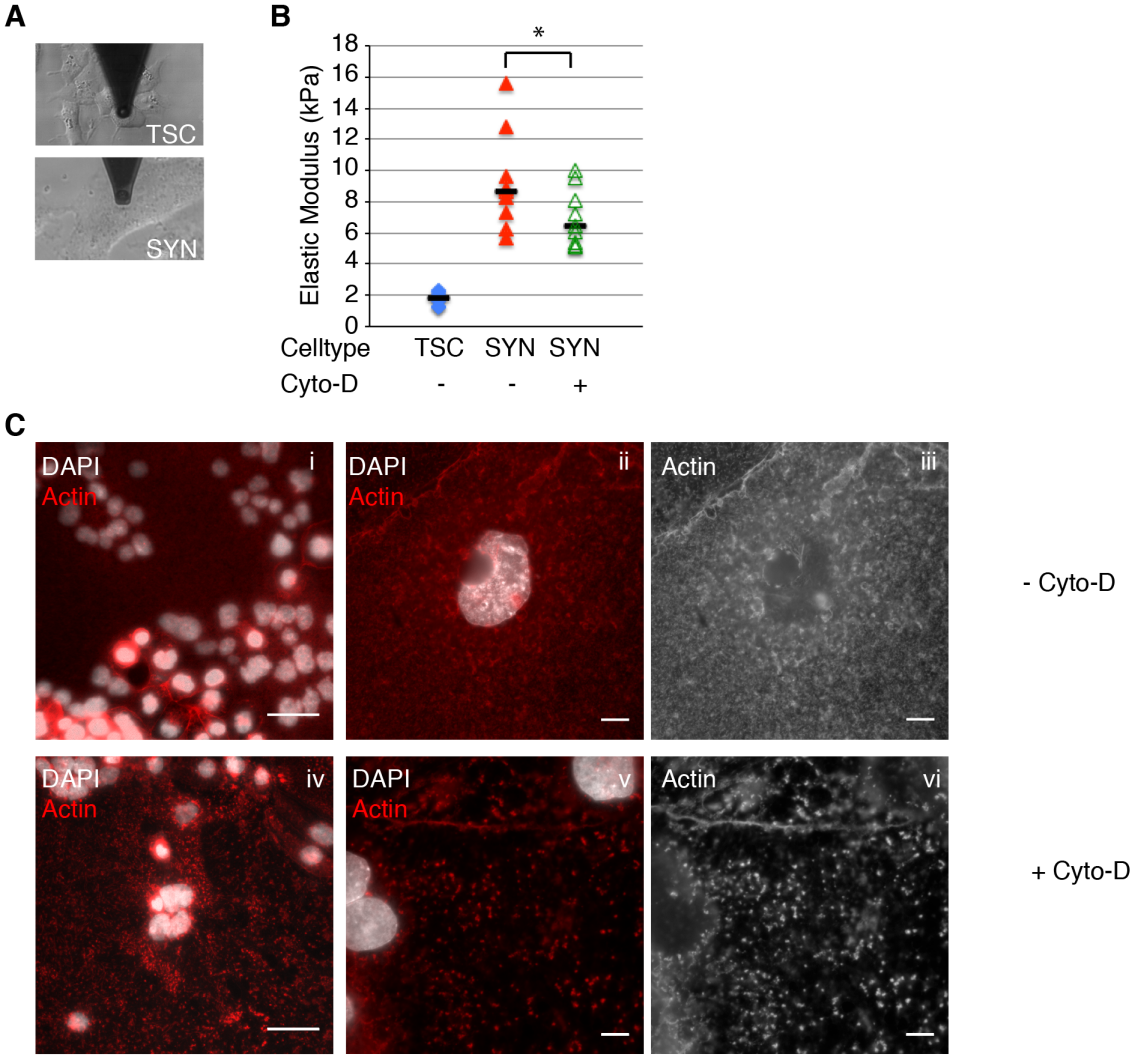
Actin cytoskeleton of murine syncytiotrophoblast (SYN) contributes to its elastic strength. A. Microrheology with an atomic force microscope was used to measure elastic strength of mouse trophoblast stem cells (TSC) and SYN. Photos depict microscopic cantilever positioned above cultured live cells prior to measurement. **B.** The elastic modulus (Young's modulus) of SYN is significantly higher than that of TSC (p = 4.7×10^−5^ by Student's T-test). Elastic modulus of SYN was measured in the exact same spot prior to and after treatment with Cyto-D for 40–60 min. Disruption of the actin cytoskeleton with Cyto-D significantly decreased the elastic modulus of SYN (p = 0.001 by Student's T-test). Bars represent median values. Graph is based on three independent experiments performed in triplicate. **C.** Immunofluorescence images of the actin (red) in mSYN show that the characteristic actin meshwork (i–iii) is disrupted by 1 hr treatment with Cyto-D (iv–vi). Nuclei are shown in white. Bars in panels i and iv are 50 um. Panels ii–iii and v–vi are representative close-up images of untreated and Cyto-D treated mSYN, respectively. Panels iii and vi show just the actin channel of ii and v respectively. Aggregation of microfilaments in distinct puncta are observed upon treatment. Bars = 10 um.

Murine TSCs were plated onto chambered glass slides and differentiated for five days. Trophoblasts were probed with a polystyrene bead (5 um diameter) mounted to a cantilever, whose deflection was detected optically (Fig. 3A). Differences in elasticity of the syncytiotrophoblasts and mTSC were obtained by oscillating the cantilever and measuring the elastic response (see Methods for details). The Young's modulus of elasticity of the cell cortex was then calculated from a Hertzian mechanics model. The median elasticity of syncytiotrophoblasts and mTSCs differed 4.8-fold (8.6 kPa versus 1.8 kPa) (Fig. 3B); a difference that was statistically significant (p = 4.7×10^−5^ by Student's T-test). The elasticity of mTSCs was within the expected range for mononuclear cells; for example, human embryonic stem cells have an elastic modulus of 1.2 kPA, while fibroblasts are typically stiffer with an elastic modulus of 3.5 kPA [34]. While a variety of cytoskeletal elements can affect cell elasticity, we hypothesized that the unique actin cytoskeletal organization of the syncytium contributes to its higher structural rigidity. Therefore, we wanted to investigate whether actin de-polymerization decreases syncytial resistance to deformation and increases susceptibility to bacterial invasion.

### Decreased surface resistance to deformation increases susceptibility to bacterial invasion

We tested this hypothesis by measuring the elastic modulus of the syncytium before and after treatment with Cytochalasin D (Cyto-D). Cyto-D depolymerizes the actin cytoskeleton [35] and has been shown to decrease the elastic modulus in other cell types [36], [37]. For each independent experiment, three distinct spots of syncytium were measured by AFM prior to treatment; Cyto-D was then added to the culture coverslip for 40–60 minutes, and measurements were repeated in those exact spots. Treatment of murine syncytiotrophoblasts for 40–60 min with Cyto-D significantly decreased the median elastic modulus by 25% (p = 0.001 by Student's T-test) (Fig. 3B). Others have shown that treatment of fibroblasts with Cyto-D can decrease cell stiffness by up to 50% [38], [39]. Decreased elastic modulus in the syncytium was accompanied by consistent morphological changes (Fig. 3C). The thick meshwork of smaller actin filaments was replaced by discrete puncta of actin aggregates. The modest effect of Cyto-D on the elastic modulus of the syncytium suggests that other cellular elements are important for maintaining syncytial stiffness. Colchicine, a microtubule polymerization inhibitor, did not have a significant effect (data not shown) on syncytial elasticity, implying yet other cytoskeletal or membrane features contribute to the tissue's rigidity.

Next, we tested whether disruption of the cortical actin network in syncytiotrophoblasts resulted in higher rates of bacterial infection. We chose to investigate the effect of Cyto-D on syncytial infection via cell-to-cell spread because *L. monocytogenes* travels inside of leukocytes in maternal blood [40], [41]. Fetal syncytiotrophoblasts are bathed in maternal blood. Hence, the syncytium comes in contact with infected maternal cells rather than extracellular bacteria. Syncytiotrophoblasts were treated with Cyto-D for 40–60 minutes, washed with PBS to remove Cyto-D, and co-incubated with infected murine macrophages in the presence of gentamicin. In this setup actin was disrupted only in the recipient trophoblast cells while bacterial protrusions formed normally in the donor macrophages. Because the physical force of a protrusion is enough for bacterial spread into an adjacent cell [42], this system allowed us to investigate the defensive role of the syncytial actin network without impairment of protrusion formation in the donor cell.

**Figure 4:**
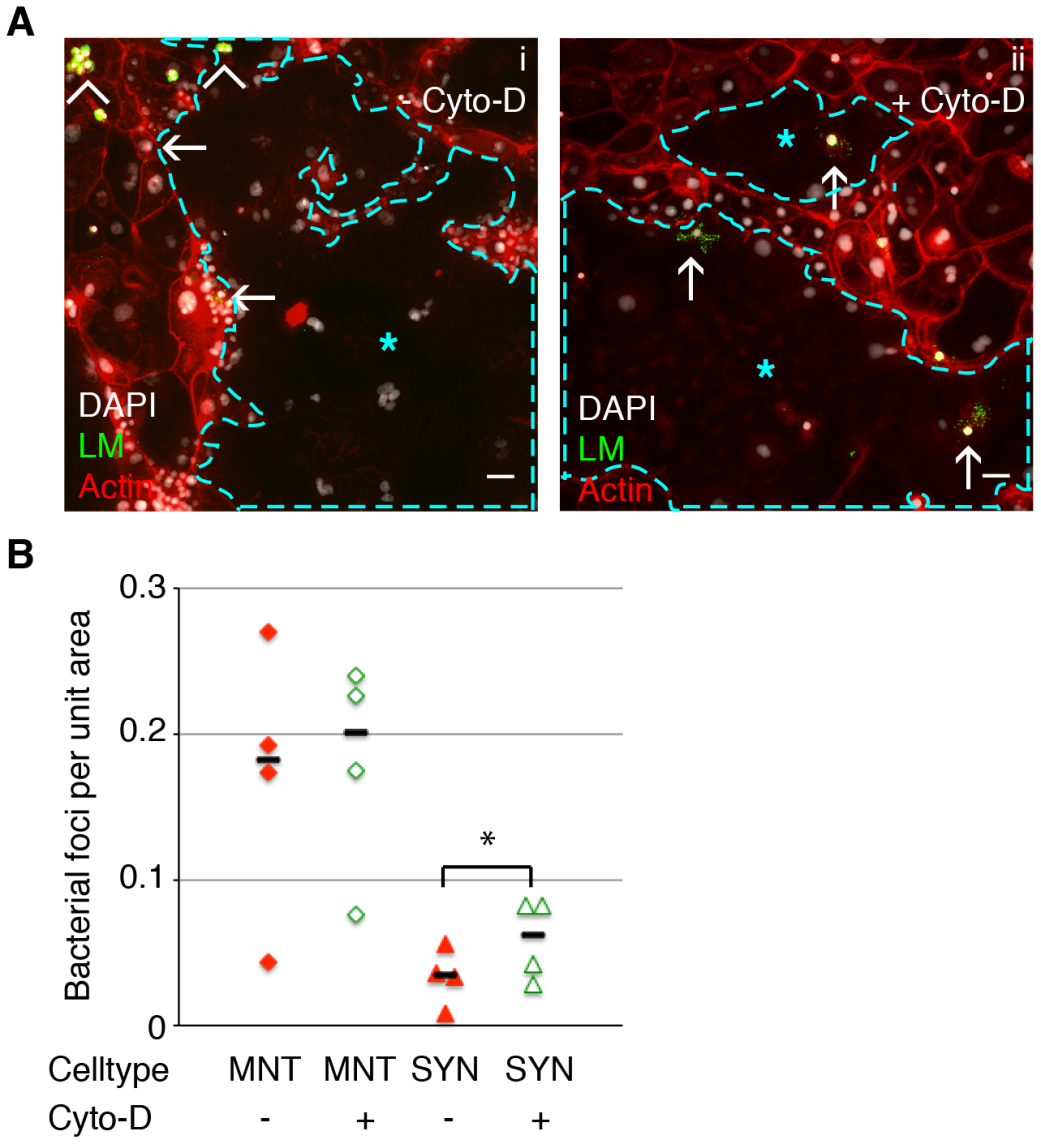
Cell-to-cell spread of *L. monocytogenes* (LM) into mouse syncytiotrophoblast (SYN) is enhanced by syncytial actin network disruption. Untreated and Cyto-D treated SYN was incubated with LM-infected murine macrophages and foci of bacterial spread were quantified. Bacterial foci were included in the analysis when multiple bacteria were observed unbounded by the outline of a macrophage membrane; many such foci were surrounded by actin clouds. **A.** Panel i: Representative immunofluorescence image of untreated differentiated TSCs shows bacterial infection of mononuclear trophoblasts (MNT) versus SYN. SYN is outlined and marked by blue star. Panel ii: One hour of Cyto-D treatment increases the number of bacterial foci in SYN. Arrowheads indicate adherence of infected macrophages to trophoblasts; arrows indicate spread events. Actin is shown in red, nuclei in blue, LM in green. Bars = 50 um. **B.** Quantification of bacterial foci per unit area in untreated versus Cyto-D treated SYN at 5 hours p.i. with macrophages. Each data point represents the average of bacterial foci in ten random fields (20×) with at least 50% syncytium. Treatment with Cyto-D significantly increases infection of SYN via cell-to-cell spread (p = 0.03 by Student's T-test); treatment does not significantly change infection of MNT (p = 0.33). Bars represent median.

In most cell lines cell-to-cell spread begins as early as 4 hours p.i. [43], [44]. Therefore we determined bacterial spread from macrophages into syncytiotrophoblasts at 5 hours p.i. by quantifying the number of bacterial foci in syncytiotrophoblasts (Fig. 4A, B). A bacterial focus was included in the analysis when multiple bacteria were observed overlying syncytiotrophoblasts unbounded by the outline of a macrophage membrane; many such foci were surrounded by actin clouds. The number of bacterial foci was 2-fold higher in Cyto-D–treated syncytiotrophoblasts in comparison to untreated controls, (p = 0.03 by Student's T-test) (Fig. 4B). In mononuclear trophoblasts Cyto-D removed stress fibers as well, but in contrast to syncytiotrophoblasts Cyto-D treatment did not significantly increase invasion into mononuclear trophoblast cells (p = 0.33 by Student's T-test). In order to test whether these findings are relevant in humans we turned to primary human placental organ cultures [20].

### Cytochalasin D treatment increases bacterial invasion of primary human placental organ cultures

**Figure 5:**
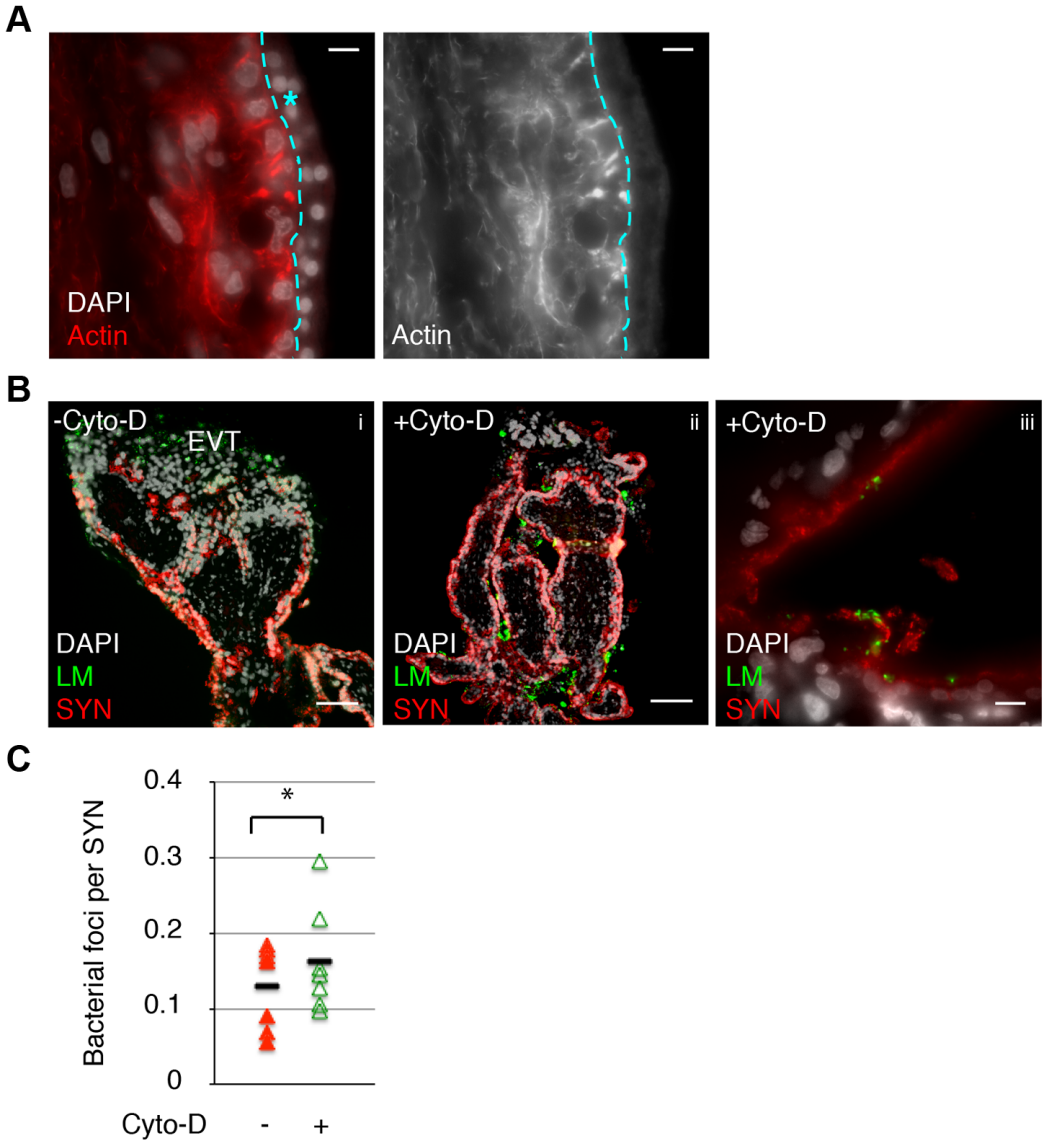
Cell-to-cell spread of *L. monocytogenes* (LM) into human syncytiotrophoblast (SYN) is enhanced by syncytial actin network disruption. A. Immunofluorescence image of sectioned primary human placenta showing diffuse actin structure in the syncytium (outlined and marked by blue star.) Bar = 10 um. **B.** Untreated and 1 hr Cyto-D treated human placental organ cultures were incubated with LM-infected macrophages and foci of spread observed and quantified. Panels i and ii show representative images showing cell-to-cell spread occurs almost exclusively at the extravillous trophoblasts (EVT) in untreated placenta; Cyto-D treatment increases incidence of spread into SYN. Bar = 100 um. Panel iii shows representative image of bacterial presence in the syncytium in Cyto-D treated placenta. Bar = 10 um. LM is shown in green, nuclei in white, SYN (b-hCG staining) is shown in red. **C.** Quantification of cell-to-cell spread into SYN. Each data point represents average co-localization of LM with b-hCG from ten microscopic fields (10×); bar represents median, graph is based on seven independent experiments. Cyto-D treatment significantly increases bacterial cell-to-cell spread into placental syncytium (p = 0.04 by Student's T-test).

It is not possible to measure the elastic strength of the human syncytium because we cannot maintain primary human syncytiotrophoblasts on glass coverslips—they require a gelatinous extracellular substratum. However, the actin cytoskeletal structure of the syncytium in primary human placental organ cultures is similar to mouse syncytiotrophoblasts (compare Fig. 5A and 2B). Given the results in the mouse syncytium, we reasoned that decreased elastic modulus of human syncytium due to Cyto-D treatment could influence its susceptibility to bacterial invasion as well. Placental organ cultures were treated with Cyto-D for 40–60 minutes, washed with PBS to remove Cyto-D, and subsequently co-cultured with *L. monocytogenes*-infected human macrophages in the presence of Gentamicin to eliminate extracellular bacteria. Infection of the syncytium was quantified microscopically by bacterial co-localization with the beta-subunit of human chorionic gonadotropin (b-hCG), a syncytial marker at 24 hours p.i. (Fig. 5B) [20]. Cyto-D treatment increased the number of bacteria co-localizing with b-hCG by 1.4-fold, a difference that was statistically significant (Fig. 5C) (p = 0.04 by Student's T-test).

## Discussion

We adapted the mouse system of differentiated mTSCs to study placental defenses against infection. We show that multinucleated fused syncytiotrophoblasts have a greater elastic modulus than mononuclear trophoblasts, and that actin contributes to this phenotype. We present evidence that disruption of the actin cytoskeleton decreases the elastic modulus of syncytiotrophoblasts and increases bacterial spread into the syncytium in both mouse and human. Taken together, these findings suggest that the biophysical properties of the syncytium, a tissue unique to the placenta, may contribute to host defense mechanisms and protect the fetus.

The cytoskeletal organization of the syncytium is characterized by an open lattice-like network of microtubules oriented in parallel to the syncytial surface, which support an apparently disordered mesh of actin microfilaments [25], [45], [46]. Our immunofluorescence microscopy showed such a diffuse actin and microtubule structure (microtubule data not shown) throughout the cytoplasm in both murine and primary human syncytiotrophoblasts [26]. Interestingly, erythrocytes have a similarly disorganized mesh of short actin fragments, which is necessary to withstand the shear forces they experience in the bloodstream [47]. The cytoskeletal organization of the syncytium may have evolved to withstand shear forces as well, since it is in direct contact with large volumes of maternal blood.

The Young's modulus of elasticity was 4.8-fold higher in syncytiotrophoblasts in comparison to mononuclear mTSCs. Interestingly, small differences in the elastic modulus of different cell types or extracellular matrices have been shown to correlate with human disease states. Dulinska et al., demonstrated that the Young's modulus in erythrocytes from patients with hemolytic anemia due to hereditary spherocytosis, thalassemia, or glucose-6-phophate dehydrogenase deficiency is 1.5 to 3.5-fold greater than the Young's modulus of normal erythrocytes [48]. A 5-fold increase in stiffness of leukemic cells correlates with clinical symptoms of leucostasis [49], and a 2 to 4-fold increase in the stiffness of extracellular matrix leads to a small but significant increase in endothelial permeability and leukocyte transmigration, a process that occurs with aging and contributes to the pathogenesis of atherosclerosis [50].

We investigated whether the elastic properties of the syncytium correlate with susceptibility to infection. *L. monocytogenes* can infect non-phagocytic host cells by two mechanisms: direct internalin-mediated invasion and receptor-independent cell-to-cell spread. During cell-to-cell spread, *L. monocytogenes* propels itself in the cytosol of the donor cell into membrane protrusions (listeriapods), which invaginate into and are taken up by neighboring cells. The force applied to the recipient cell by the listeriapod is estimated to be 0.03–0.3 nN [51], and has been found to be sufficient for bacterial uptake in cultured cells [42]. The stiffness of the recipient cell may correlate with its susceptibility to bacterial transmission: *L. monocytogenes* secretes internalin C, which promotes cell-to-cell spread by binding to the cytosolic adaptor protein Tuba, which in turn leads to slackened cell-cell junctions and decreased cortical tension [52].

We used Cyto-D to disrupt the actin cytoskeleton of syncytiotrophoblasts, which decreased their elastic modulus by 25%. In our experimental setup, Cyto-D was added to the placental culture medium to disrupt actin structures and subsequently washed out before addition of untreated donor macrophages, allowing listeriapods in the donor cell to occur normally. Actin structures appeared to return to their original diffuse meshwork configuration over the course of the infection period (data not shown), implying actin dynamics resumed with the removal of the drug. Exposure to Cyto-D led to a small increase in infection via cell-to-cell spread in both of our model systems: murine and human syncytiotrophoblasts. Our findings suggest two important conclusions: 1) the syncytial horizontal integrity is actively maintained by continuous actin assembly; and, 2) this structure is not permissive to the short-term perpendicular rearrangements engineered by listeriapods.

We admit that the effect of Cyto-D treatment on elasticity and infection is small. Unfortunately, we were unable to decrease the elastic modulus even further by either increasing the dose or prolonging the exposure to Cyto-D, because of drug induced cytotoxicity (data not shown). Thus we were unable to decrease the elastic modulus of syncytiotrophoblasts to a range comparable with mononuclear cells and we were also unable to maintain these conditions for the entire time of the experiment, which is dictated by the kinetics of cell-to-cell spread. These experimental limitations are one of the possible reasons why Cyto-D treatment led to only a small increase in infection. On the other hand, it is plausible that additional mechanisms influence the susceptibility of the syncytium to infection. Further, we cannot discount the possibility that Cyto-D has unknown side effects on placental cells that may affect their susceptibility to infection as well.

Nevertheless, we speculate that syncytial elasticity influences its susceptibility to infection in vivo. Others have found that organization of the actin cytoskeleton can contribute to resistance against pathogen invasion. For instance, plant cells resist penetration by fungal pathogens via actin cytoskeletal reorganization [53]. On the other hand, it has been shown recently that some mammalian pathogens have evolved strategies to subvert the defenses of the actin cytoskeleton. The protozoan pathogen *Toxoplasma gondii* uses the virulence determinant Toxofilin to loosen the local host cell actin meshwork to facilitate invasion [54]. Pretreatment of host cells with jasplakinolide, which stabilizes actin filaments, renders them refractory to subsequent parasite entry [55]. However, Toxofilin is apparently inadequate to the task of syncytial actin rearrangement, as *T. gondii* are significantly inhibited from syncytial invasion [21].

The long-held view of the placenta as an immune compromised organ is slowly being eroded by the discovery of remarkable, unique innate defense mechanisms at the maternal-fetal interface. It appears that multiple forces have to coalesce to damage this barrier: placentas from preterm labor and congenital infections are often colonized by multiple pathogens [56], [57], and bacterial products synergize with viral infection to trigger preterm labor in the mouse model [58], [59]. Damage to syncytiotrophoblasts by *Plasmodium falciparum* [60], [61] or *Trypanosoma cruzi* [62] could lead to increased transmission rates of co-pathogens such as HIV [63]. Importantly, host genetic factors may also predispose to preterm labor triggered by bacterial products [12]. We suggest that even small changes in the integrity of the syncytial barrier may predispose the maternal-fetal interface to infections that lead to pregnancy complications, fetal damage and death.

In summary, the placental syncytium is a unique structure that arose independently in many different mammals with hemochorial placentation [64]. Its critical role in the maintenance of healthy human pregnancy has been documented in terms of hormone production and proper nutrient and waste exchange. The contribution of its remarkable biophysical properties to resistance against pathogen invasion warrants further study.

## Methods

### Ethics statement

This study was conducted according to the principles expressed in the Declaration of Helsinki. The study was approved by the Institutional Review Board at the University of California, San Francisco, where all experiments were performed (H497-00836-28). All patients provided written informed consent for the collection of samples and subsequent analysis.

### Human tissue collection and culture

All chemicals were purchased from Sigma-Aldrich unless otherwise stated. Placentas from elective terminations of pregnancy (gestational age 4 to 8 weeks) were collected and prepared as previously described [20]. Briefly, fragments from the surface of the placenta were dissected into 1–3 mm tree-like villi, placed on Matrigel (BD Biosciences, San Jose, CA)-coated Transwell filters (Millipore, Bedirica, MA, 30-mm diameter, 0.4 um pore size) and cultured in Dulbecco's modified Eagle's medium-F12 medium (DMEM-F12; 1∶1, vol/vol) supplemented with 20% fetal bovine serum (FBS, Fisher Scientific), 1% L-glutamine and 1% penicillin/streptomycin (Invitrogen, Carlsbad, CA).

### Mouse trophoblast culture and syncytialization

CellStart Humanized Substrate for Cell Culture (Cell Therapy Systems) was used for culture of mouse trophoblast stem cells (mTSC). Cells were plated onto dishes pre-coated with CellStart diluted at 1∶20 with PBS, and maintained in RPMI-1640 with 20% FBS, 1% sodium pyruvate, 100 uM b-mercaptoethanol, 1% L-glutamine, 1% penicillin/streptomycin. FGF4 (25 ng/mL) and Heparin (1 ug/mL) were added fresh to media each time cells were thawed or split.

For differentiation into syncytiotrophoblasts, mTSC were seeded onto coverslips pre-coated with 0.1% gelatin in 24-well dishes at 35,000 cells/well. Cells were maintained in RPMI-1640 with 20% FBS, 1% sodium pyruvate, 100 uM b-mercaptoethanol, 1% L-glutamine, 1% penicillin/streptomycin, and 10 uM U0126 (MEK inhibitor, Pierce Biotechnology, Rockford, IL) for 5 days. Fresh media containing MEK inhibitor was added to the culture every 2 days.

### Pathogen strains and growth conditions

The wild type strain of *L. monocytogenes* used in this study is 10403S [28]. *L. monocytogenes* with murinized InlA replacing WT InlA was a gift from Dr. Manuel Amieva [32]. For infections, bacteria were grown overnight to stationary phase in BHI (Brain Heart Infusion broth) at 30°C and washed once with PBS before dilution and infection.

### *L. monocytogenes* infection of mouse trophoblasts

For mTSC infection cells were incubated in antibiotic-free medium for 1 hr before infection. 3×10^6^ bacteria/mL were added for 60 minutes; cells were washed once with PBS and fresh media with gentamicin (50 ug/mL) was added. At indicated times, cells were lysed, aliquots were plated on BHI agar plates, and CFU were enumerated. Five-day differentiated murine syncytiotrophoblasts (mSYN) were infected under the same conditions. At indicated times, cells were fixed, stained with phalloidin and polyclonal rabbit *Listeria* O antiserum and examined microscopically. Green fluorescence intensity in six random fields per area of syncytiotrophoblast versus mononuclear trophoblast was determined. For infection of mSYN via cell-to-cell spread from macrophages, J774 cell line (ATCC TIB-67) was infected with 3×10^6^ bacteria/mL (MOI 3) for 60 minutes. Concurrently, mSYN was incubated with antibiotic-free media +/− Cyto-D (10 uM) for 1 hr. Macrophages were washed 1× with PBS, gently scraped off the dishes and resuspended in mouse trophoblast media containing gentamicin (50 ug/mL). Infected macrophages were added to mSYN cultured on coverslips at 100,000 cells/well.

### *L. monocytogenes* infection of placental explants by cell-to-cell spread

Placental explants were infected via cell-to-cell spread from human macrophage-like U937 cells (ATCC 1593.2) as previously described [20]. Briefly, U937 cells were grown in RPMI-1640 (UCSF Cell Culture Facility) containing 4500 mg/L glucose, 10% FBS and 1% penicillin/streptomycin (Invitrogen). Forty-eight hrs prior to infection, cells were differentiated by addition of phorbol 12-myristate 13-acetate (PMA; concentration 18 nM) to the medium. On the day of infection, cells were incubated with antibiotic-free medium for 1 hr and subsequently infected with *L. monocytogenes* for 1 hr at an MOI of 3. Concurrently, explants were incubated with antibiotic-free media +/− Cyto-D (10 uM) for 1 hr, and subsequently washed 3× with PBS. U937 cells were washed once with PBS and lifted from culture plates by incubation in ice cold PBS without divalent cations and gentle scraping. U937 cells were resuspended in explant medium containing 50 ug/ml gentamicin, and 1×10^6^ cells per transwell were added to the explants.

### Immunofluorescence

Human placental explants were fixed in 3% paraformaldehyde, passed through a sucrose gradient and snap-frozen in OCT (Ted Pella, Redding, CA). Histological slicing was performed on a Hacker-Slee cryostat. Glass slides with sections were incubated in acetone, soaked in blocking solution (1% bovine serum albumin (BSA) in PBS), then incubated with primary antibodies, rinsed in PBS, incubated with secondary antibodies, and affixed over Vectashield mounting medium with DAPI (Vector Laboratories, Burlingame, CA).

Mouse trophoblast cultures were fixed in 4% paraformaldehyde, blocked and permeabilized in 1% BSA and 0.1% Triton-X100, then stained as described above in BSA/TritonX-100/PBS solution.

Primary antibodies: polyclonal rabbit *Listeria* O antiserum (1∶1000, BD Biosciences, San Jose, CA), monoclonal mouse anti-human b-hCG (1∶500, Neomarkers, Fremont, CA, clone SPM105.) Secondary antibodies: Alexa Fluor 594 goat anti-mouse IgG (1∶500, Invitrogen), Alexa Fluor 488 and 594 goat anti-rabbit IgG (1∶1000 & 1∶500, Invitrogen). Alexa Fluor 594 – conjugated phalloidin (1∶100, Invitrogen) was used to stain for actin.

Slides were viewed using an inverted TE2000-E microscope (Nikon, Tokyo, Japan) equipped with a 12-bit cooled CCD camera (Q imaging, Surrey, Canada). Images were collected using Simple PCI software (Hamamats, Sewickley, PA).

### Atomic force microscopy

Elasticity measurements were recorded at room temperature with a modified commercial AFM (Bruker Bioscope Catalyst). Samples were mounted on a Zeiss Observer Z1 microscope. Optical images were acquired with an EM CCD camera (Andor Ixon+). Cantilevers were prepared by gluing (Norland 61) polystyrene beads (diameter 5 um) to a tipless un-coated cantilever (Veeco, customized MLCT, cantilever with a nominal spring constant of 0.01 N/m); the cantilevers' spring constants were individually determined by the thermal vibration method before each measurement [65]. To monitor the elasticity of the syncytium membranes and stem cells, the polystyrene bead was moved in contact with the sample at a constant force of 0.5 nN in average. To measure the elasticity, a sinusoid oscillation with amplitude of 20 nm was applied to the cantilever vertically at 3 Hz. The amplitude and phase shift of the oscillatory cantilever deflection caused by viscoelasticity of the sample was detected with a lock-in amplifier (Signal Recovery 7270). The time constant of the lock-in amplifier was 1 second with the sensitivity at 500 mV. The elasticity measurements were recorded with a custom LabView program together with a National Instrument DAQcard (PCI-6229) and an electronic signal filter (Krohn-hite 3364). For the analysis of the elastic modulus, calculations were based on the common Hertz model extended by Tu and Chen models [66]. The effect of Cyto-D on syncytial elasticity was observed by measuring the same spot of syncytium before and after 40–60 minutes of treatment.

### Image processing

Images were prepared using ImageJ (RSB, Bethesda, MD). JaCoP plugin for ImageJ was used for quantifying co-localization. Image files were blinded, and Manders' coefficients [67] were calculated, with individually set threshold for each channel in each image. The green channel (LM) threshold was set to exclude auto-fluorescence and background, but include all bacteria. Red fluorescence (b-hCG) threshold was set to include only the outer-most layer of cells on explant (syncytium) and exclude background.

## References

[1] Goldenberg RL, Hauth JC, Andrews WW (2000) Intrauterine infection and preterm delivery. N Engl J Med 342: 1500–1507. 10816189 10.1056/NEJM200005183422007

[2] Beck S, Wojdyla D, Say L, Betran AP, Merialdi M, et al (2010) The worldwide incidence of preterm birth: a systematic review of maternal mortality and morbidity. Bull World Health Organ 88: 31–38. 20428351 10.2471/BLT.08.062554 PMC2802437

[3] Robbins JR, Bakardjiev AI (2012) Pathogens and the placental fortress. Curr Opin Microbiol 15: 36–43. 22169833 10.1016/j.mib.2011.11.006 PMC3265690

[4] Pouillot R, Hoelzer K, Jackson KA, Henao OL, Silk BJ (2012) Relative risk of listeriosis in Foodborne Diseases Active Surveillance Network (FoodNet) sites according to age, pregnancy, and ethnicity. Clin Infect Dis 54 (Suppl 5) S405–410. 22572661 10.1093/cid/cis269

[5] Vital signs: Listeria illnesses, deaths, and outbreaks–United States, 2009-2011. MMWR Morb Mortal Wkly Rep 62: 448–452. PMC4604984 23739339

[6] Mylonakis E, Paliou M, Hohmann EL, Calderwood SB, Wing EJ (2002) Listeriosis during pregnancy: a case series and review of 222 cases. Medicine (Baltimore) 81: 260–269. 12169881 10.1097/00005792-200207000-00002

[7] Siegman-Igra Y, Levin R, Weinberger M, Golan Y, Schwartz D, et al (2002) *Listeria monocytogenes* infection in Israel and review of cases worldwide. Emerg Infect Dis 8: 305–310. 11927029 10.3201/eid0803.010195 PMC3369577

[8] Benshushan A, Tsafrir A, Arbel R, Rahav G, Ariel I, et al (2002) *Listeria* infection during pregnancy: a 10 year experience. Isr Med Assoc J 4: 776–780. 12389339

[9] Gellin BG, Broome CV, Bibb WF, Weaver RE, Gaventa S, et al (1991) The epidemiology of listeriosis in the United States–1986. Listeriosis Study Group. Am J Epidemiol 133: 392–401. 1899779 10.1093/oxfordjournals.aje.a115893

[10] Schuchat A, Lizano C, Broome CV, Swaminathan B, Kim C, et al (1991) Outbreak of neonatal listeriosis associated with mineral oil. Pediatr Infect Dis J 10: 183–189. 2041663 10.1097/00006454-199103000-00003

[11] Notermans S, Dufrenne J, Teunis P, Chackraborty T (1998) Studies on the risk assessment of *Listeria monocytogenes* . J Food Prot 61: 244–248. 9708290 10.4315/0362-028x-61.2.244

[12] Cha J, Bartos A, Egashira M, Haraguchi H, Saito-Fujita T, et al (2013) Combinatory approaches prevent preterm birth profoundly exacerbated by gene-environment interactions. J Clin Invest 123: 4063–4075. 23979163 10.1172/JCI70098 PMC3754274

[13] Erlebacher A (2013) Immunology of the Maternal-Fetal Interface. Annu Rev Immunol 10.1146/annurev-immunol-032712-100003 23298207

[14] Zeldovich VB, Bakardjiev AI (2012) Host defense and tolerance: unique challenges in the placenta. PLoS Pathog 8: e1002804. 22912572 10.1371/journal.ppat.1002804 PMC3415450

[15] Medawar PB (1953) Some immunological and endocrinological problems raised by the evolution of viviparity in vertebrates. Symp Soc Exp Biol 7: 320–338.

[16] Mor G, Cardenas I, Abrahams V, Guller S (2011) Inflammation and pregnancy: the role of the immune system at the implantation site. Ann N Y Acad Sci 1221: 80–87. 21401634 10.1111/j.1749-6632.2010.05938.x PMC3078586

[17] Delorme-Axford E, Donker RB, Mouillet JF, Chu T, Bayer A, et al (2013) Human placental trophoblasts confer viral resistance to recipient cells. Proc Natl Acad Sci U S A 110: 12048–12053. 23818581 10.1073/pnas.1304718110 PMC3718097

[18] Maltepe E, Bakardjiev AI, Fisher SJ (2010) The placenta: transcriptional, epigenetic, and physiological integration during development. J Clin Invest 120: 1016–1025. 20364099 10.1172/JCI41211 PMC2846055

[19] Benirschke K, Kaufmann P, Baergen RN (2006) Pathology of the Human Placenta.

[20] Robbins JR, Skrzypczynska KM, Zeldovich VB, Kapidzic M, Bakardjiev AI (2010) Placental syncytiotrophoblast constitutes a major barrier to vertical transmission of *Listeria monocytogenes* . PLoS Pathog 6: e1000732. 20107601 10.1371/journal.ppat.1000732 PMC2809766

[21] Robbins JR, Zeldovich VB, Poukchanski A, Boothroyd JC, Bakardjiev AI (2012) Tissue barriers of the human placenta to infection with *Toxoplasma gondii* . Infect Immun 80: 418–428. 22083708 10.1128/IAI.05899-11 PMC3255695

[22] Koi H, Zhang J, Makrigiannakis A, Getsios S, MacCalman CD, et al (2002) Syncytiotrophoblast is a barrier to maternal-fetal transmission of herpes simplex virus. Biol Reprod 67: 1572–1579. 12390890 10.1095/biolreprod.102.004325

[23] Aplin JD, Jones CJ, Harris LK (2009) Adhesion molecules in human trophoblast - a review. I. Villous trophoblast. Placenta 30: 293–298. 19131106 10.1016/j.placenta.2008.12.001

[24] Bonazzi M, Cossart P (2011) Impenetrable barriers or entry portals? The role of cell-cell adhesion during infection. J Cell Biol 195: 349–358. 22042617 10.1083/jcb.201106011 PMC3206337

[25] Ockleford CD, Wakely J, Badley RA (1981) Morphogenesis of human placental chorionic villi: cytoskeletal, syncytioskeletal and extracellular matrix proteins. Proc R Soc Lond B Biol Sci 212: 305–316. 6115395 10.1098/rspb.1981.0041

[26] Choi HJ, Sanders TA, Tormos KV, Ameri K, Tsai JD, et al (2013) ECM-Dependent HIF Induction Directs Trophoblast Stem Cell Fate via LIMK1-Mediated Cytoskeletal Rearrangement. PLoS One 8: e56949. 23437279 10.1371/journal.pone.0056949 PMC3578927

[27] Tanaka S, Kunath T, Hadjantonakis AK, Nagy A, Rossant J (1998) Promotion of trophoblast stem cell proliferation by FGF4. Science 282: 2072–2075. 9851926 10.1126/science.282.5396.2072

[28] Bishop DK, Hinrichs DJ (1987) Adoptive transfer of immunity to *Listeria monocytogenes*. The influence of in vitro stimulation on lymphocyte subset requirements. J Immunol 139: 2005–2009. 3114382

[29] Lecuit M, Nelson DM, Smith SD, Khun H, Huerre M, et al (2004) Targeting and crossing of the human maternofetal barrier by *Listeria monocytogenes*: role of internalin interaction with trophoblast E-cadherin. Proc Natl Acad Sci U S A 101: 6152–6157. 15073336 10.1073/pnas.0401434101 PMC395938

[30] Bakardjiev AI, Stacy BA, Fisher SJ, Portnoy DA (2004) Listeriosis in the pregnant guinea pig: a model of vertical transmission. Infect Immun 72: 489–497. 14688130 10.1128/IAI.72.1.489-497.2004 PMC343973

[31] Lecuit M, Dramsi S, Gottardi C, Fedor-Chaiken M, Gumbiner B, et al (1999) A single amino acid in E-cadherin responsible for host specificity towards the human pathogen *Listeria monocytogenes* . EMBO J 18: 3956–3963. 10406800 10.1093/emboj/18.14.3956 PMC1171471

[32] Wollert T, Pasche B, Rochon M, Deppenmeier S, van den Heuvel J, et al (2007) Extending the host range of *Listeria monocytogenes* by rational protein design. Cell 129: 891–902. 17540170 10.1016/j.cell.2007.03.049

[33] Behm RJ, Hosler W, Ritter E, Binning G (1986) Correlation between domain boundaries and surface steps: A scanning-tunneling-microscopy study on reconstructed Pt(100). Phys Rev Lett 56: 228–231. 10033130 10.1103/PhysRevLett.56.228

[34] Hammerick KE, Huang Z, Sun N, Lam MT, Prinz FB, et al (2011) Elastic properties of induced pluripotent stem cells. Tissue Eng Part A 17: 495–502. 20807017 10.1089/ten.tea.2010.0211 PMC3052278

[35] Sampath P, Pollard TD (1991) Effects of cytochalasin, phalloidin, and pH on the elongation of actin filaments. Biochemistry 30: 1973–1980. 1899622 10.1021/bi00221a034

[36] Nagayama K, Nagano Y, Sato M, Matsumoto T (2006) Effect of actin filament distribution on tensile properties of smooth muscle cells obtained from rat thoracic aortas. J Biomech 39: 293–301. 16321631 10.1016/j.jbiomech.2004.11.019

[37] Moreno-Flores S, Benitez R, Vivanco M, Toca-Herrera JL (2010) Stress relaxation and creep on living cells with the atomic force microscope: a means to calculate elastic moduli and viscosities of cell components. Nanotechnology 21: 445101. 20921592 10.1088/0957-4484/21/44/445101

[38] Wakatsuki T, Schwab B, Thompson NC, Elson EL (2001) Effects of cytochalasin D and latrunculin B on mechanical properties of cells. J Cell Sci 114: 1025–1036. 11181185 10.1242/jcs.114.5.1025

[39] Rotsch C, Radmacher M (2000) Drug-induced changes of cytoskeletal structure and mechanics in fibroblasts: an atomic force microscopy study. Biophys J 78: 520–535. 10620315 10.1016/S0006-3495(00)76614-8 PMC1300659

[40] Drevets DA, Jelinek TA, Freitag NE (2001) *Listeria monocytogenes*-infected phagocytes can initiate central nervous system infection in mice. Infect Immun 69: 1344–1350. 11179297 10.1128/IAI.69.3.1344-1350.2001 PMC98026

[41] Bakardjiev AI, Theriot JA, Portnoy DA (2006) *Listeria monocytogenes* traffics from maternal organs to the placenta and back. PLoS Pathog 2: e66. 16846254 10.1371/journal.ppat.0020066 PMC1483233

[42] Monack DM, Theriot JA (2001) Actin-based motility is sufficient for bacterial membrane protrusion formation and host cell uptake. Cell Microbiol 3: 633–647. 11553015 10.1046/j.1462-5822.2001.00143.x

[43] Robbins JR, Barth AI, Marquis H, de Hostos EL, Nelson WJ, et al (1999) *Listeria monocytogenes* exploits normal host cell processes to spread from cell to cell. J Cell Biol 146: 1333–1350. 10491395 10.1083/jcb.146.6.1333 PMC1785326

[44] Tilney LG, Portnoy DA (1989) Actin filaments and the growth, movement, and spread of the intracellular bacterial parasite, *Listeria monocytogenes* . J Cell Biol 109: 1597–1608. 2507553 10.1083/jcb.109.4.1597 PMC2115783

[45] Berryman M, Gary R, Bretscher A (1995) Ezrin oligomers are major cytoskeletal components of placental microvilli: a proposal for their involvement in cortical morphogenesis. J Cell Biol 131: 1231–1242. 8522586 10.1083/jcb.131.5.1231 PMC2120629

[46] King BF (1983) The organization of actin filaments in human placental villi. J Ultrastruct Res 85: 320–328. 6427473 10.1016/s0022-5320(83)90043-6

[47] Nans A, Mohandas N, Stokes DL (2011) Native ultrastructure of the red cell cytoskeleton by cryo-electron tomography. Biophys J 101: 2341–2350. 22098732 10.1016/j.bpj.2011.09.050 PMC3218374

[48] Dulinska I, Targosz M, Strojny W, Lekka M, Czuba P, et al (2006) Stiffness of normal and pathological erythrocytes studied by means of atomic force microscopy. J Biochem Biophys Methods 66: 1–11. 16443279 10.1016/j.jbbm.2005.11.003

[49] Lam WA, Rosenbluth MJ, Fletcher DA (2008) Increased leukaemia cell stiffness is associated with symptoms of leucostasis in paediatric acute lymphoblastic leukaemia. Br J Haematol 142: 497–501. 18544083 10.1111/j.1365-2141.2008.07219.x PMC7899135

[50] Huynh J, Nishimura N, Rana K, Peloquin JM, Califano JP, et al (2011) Age-related intimal stiffening enhances endothelial permeability and leukocyte transmigration. Sci Transl Med 3: 112ra122. 10.1126/scitranslmed.3002761 PMC3693751 22158860

[51] Giardini PA, Fletcher DA, Theriot JA (2003) Compression forces generated by actin comet tails on lipid vesicles. Proc Natl Acad Sci U S A 100: 6493–6498. 12738883 10.1073/pnas.1031670100 PMC164474

[52] Rajabian T, Gavicherla B, Heisig M, Muller-Altrock S, Goebel W, et al (2009) The bacterial virulence factor InlC perturbs apical cell junctions and promotes cell-to-cell spread of *Listeria* . Nat Cell Biol 11: 1212–1218. 19767742 10.1038/ncb1964 PMC2755649

[53] Hardham AR, Jones DA, Takemoto D (2007) Cytoskeleton and cell wall function in penetration resistance. Curr Opin Plant Biol 10: 342–348. 17627866 10.1016/j.pbi.2007.05.001

[54] Delorme-Walker V, Abrivard M, Lagal V, Anderson K, Perazzi A, et al (2012) Toxofilin upregulates the host cortical actin cytoskeleton dynamics, facilitating *Toxoplasma* invasion. J Cell Sci 125: 4333–4342. 22641695 10.1242/jcs.103648 PMC3516439

[55] Gonzalez V, Combe A, David V, Malmquist NA, Delorme V, et al (2009) Host cell entry by apicomplexa parasites requires actin polymerization in the host cell. Cell Host Microbe 5: 259–272. 19286135 10.1016/j.chom.2009.01.011

[56] Onderdonk AB, Delaney ML, DuBois AM, Allred EN, Leviton A (2008) Detection of bacteria in placental tissues obtained from extremely low gestational age neonates. Am J Obstet Gynecol 198: 110 e111–117. 18166321 10.1016/j.ajog.2007.05.044

[57] Pereira L, Maidji E, McDonagh S, Genbacev O, Fisher S (2003) Human cytomegalovirus transmission from the uterus to the placenta correlates with the presence of pathogenic bacteria and maternal immunity. J Virol 77: 13301–13314. 14645586 10.1128/JVI.77.24.13301-13314.2003 PMC296088

[58] Cardenas I, Mor G, Aldo P, Lang SM, Stabach P, et al (2011) Placental viral infection sensitizes to endotoxin-induced pre-term labor: a double hit hypothesis. Am J Reprod Immunol 65: 110–117. 20712808 10.1111/j.1600-0897.2010.00908.x PMC3025809

[59] Cardenas I, Means RE, Aldo P, Koga K, Lang SM, et al (2010) Viral infection of the placenta leads to fetal inflammation and sensitization to bacterial products predisposing to preterm labor. J Immunol 185: 1248–1257. 20554966 10.4049/jimmunol.1000289 PMC3041595

[60] Crocker IP, Tanner OM, Myers JE, Bulmer JN, Walraven G, et al (2004) Syncytiotrophoblast degradation and the pathophysiology of the malaria-infected placenta. Placenta 25: 273–282. 15028419 10.1016/j.placenta.2003.09.010

[61] Hromatka BS, Ngeleza S, Adibi JJ, Niles RK, Tshefu AK, et al (2013) Histopathologies, immunolocalization, and a glycan binding screen provide insights into *Plasmodium falciparum* interactions with the human placenta. Biol Reprod 88: 154. 23575149 10.1095/biolreprod.112.106195 PMC4070867

[62] Duaso J, Rojo G, Jana F, Galanti N, Cabrera G, et al (2011) *Trypanosoma cruzi* induces apoptosis in ex vivo infected human chorionic villi. Placenta 32: 356–361. 21420164 10.1016/j.placenta.2011.02.005

[63] Bulterys PL, Chao A, Dalai SC, Zink MC, Dushimimana A, et al (2011) Placental malaria and mother-to-child transmission of human immunodeficiency virus-1 in rural Rwanda. Am J Trop Med Hyg 85: 202–206. 21813835 10.4269/ajtmh.2011.10-0589 PMC3144813

[64] Dupressoir A, Lavialle C, Heidmann T (2012) From ancestral infectious retroviruses to bona fide cellular genes: role of the captured syncytins in placentation. Placenta 33: 663–671. 22695103 10.1016/j.placenta.2012.05.005

[65] Stark RW, Drobek T, Heckl WM (2001) Thermomechanical noise of a free v-shaped cantilever for atomic-force microscopy. Ultramicroscopy 86: 207–215. 11215624 10.1016/s0304-3991(00)00077-2

[66] Mahaffy RE, Park S, Gerde E, Kas J, Shih CK (2004) Quantitative analysis of the viscoelastic properties of thin regions of fibroblasts using atomic force microscopy. Biophys J 86: 1777–1793. 14990504 10.1016/S0006-3495(04)74245-9 PMC1304012

[67] Manders EM, Stap J, Brakenhoff GJ, van Driel R, Aten JA (1992) Dynamics of three-dimensional replication patterns during the S-phase, analysed by double labelling of DNA and confocal microscopy. J Cell Sci 103 (Pt 3) 857–862. 1478975 10.1242/jcs.103.3.857

